# Mineral and Phytic Acid Content as Well as Phytase Activity in Flours and Breads Made from Different Wheat Species

**DOI:** 10.3390/ijms24032770

**Published:** 2023-02-01

**Authors:** C. Friedrich. H. Longin, Muhammad Afzal, Jens Pfannstiel, Ute Bertsche, Tanja Melzer, Andrea Ruf, Christoph Heger, Tobias Pfaff, Margit Schollenberger, Markus Rodehutscord

**Affiliations:** 1 State Plant Breeding Institute, University of Hohenheim, 70599 Stuttgart, Germany; 2 Core Facility Hohenheim, University of Hohenheim, 70599 Stuttgart, Germany; 3 Consulting Firm “Einfach.Brot.machen”, 83620 Feldkirchen-Westerham, Germany; 4 Academy of German Bakery South West e.V., 70182 Stuttgart, Germany; 5 Institute of Animal Science, University of Hohenheim, 70599 Stuttgart, Germany

**Keywords:** phytase, phytic acid, minerals, FODMAP, acrylamide

## Abstract

Wheat is of high importance for a healthy and sustainable diet for the growing world population, partly due to its high mineral content. However, several minerals are bound in a phytate complex in the grain and unavailable to humans. We performed a series of trials to compare the contents of minerals and phytic acid as well as phytase activity in several varieties from alternative wheat species spelt, emmer and einkorn with common wheat. Additionally, we investigated the potential of recent popular bread making recipes in German bakeries to reduce phytic acid content, and thus increase mineral bioavailability in bread. For all studied ingredients, we found considerable variance both between varieties within a species and across wheat species. For example, whole grain flours, particularly from emmer and einkorn, appear to have higher mineral content than common wheat, but also a higher phytic acid content with similar phytase activity. Bread making recipes had a greater effect on phytic acid content in the final bread than the choice of species for whole grain flour production. Recipes with long yeast proofing or sourdough and the use of whole grain rye flour in a mixed wheat bread minimized the phytic acid content in the bread. Consequently, optimizing food to better nourish a growing world requires close collaboration between research organizations and practical stakeholders ensuring a streamlined sustainable process from farm to fork.

## 1. Introduction

Wheat (*Triticum aestivum* subsp. *aestivum*) is one of the most important staple foods worldwide and its daily consumption is part of a healthy and balanced diet for humans (i.e., planetary health diet: https://eatforum.org/eat-lancet-commission/, accessed on 29 January 2023). In addition to human nutrition, wheat is largely used as feed or to generate renewable energy and value-added products ideally improving environmental sustainability via upcycling of side products, e.g., straw, bran, etc. [1,2]. For human diets, whole grain products should be consumed first and foremost, because dietary fibers and minerals are highly concentrated in the outer layers and germ of the grains [3,4,5]. According to the WHO, over two billion people worldwide suffer from deficiencies in micronutrients such as folate, iron (Fe), zinc (Zn), and selenium (Se), known as hidden hunger [6] (https://www.who.int/nutrition/topics/ida/en/, accessed on 29 January 2023). This is particularly pronounced in regions of the world where not enough fruits, vegetables and animal products are available for a balanced diet in addition to cereals, i.e., in parts of Asia and especially in sub-Saharan African countries. In these regions, for example, the International Maize and Wheat Improvement Center CIMMYT is also active with a special wheat breeding program in which, in addition to good agronomic performance, wheat is bred for higher levels of minerals such as Fe, Zn, and Se [3]. However, even in Europe, up to 20% of the human population is estimated to be deficient in minerals [7].

Phytic acid is the main storage form of phosphate in cereals amounting up to 70% of total seed phosphate in cereals [8]. In addition, minerals such as Fe, Zn and Se are partially bound by phytic acid in a complex [8,9,10]. Monogastric animals and humans have only very low intrinsic phytase activity in their digestive tracts [8]. Thus, these minerals remain bound to phytic acid without absorption from the intestine and are largely excreted undigested, which in the case of phosphate is also of importance for the environment. Thus, the availability of minerals is a nutritional issue for humans and animals as well as an issue of environmental and resource protection. The latter issue has long been the subject of scientific research [11].

Ideally, a cereal should have many minerals, low phytic acid content and high activity of the cereal’s own phytase as shown in a comprehensive review article by Brinch-Pedersen et al. [8]. However, wheat grain with increased plant intrinsic phytase activity did not increase phytic acid degradation in pigs and chicken when compared with a standard wheat [12,13]. This may be one reason why microbial phytase is widely used as feed additive in animal nutrition even when grains with high plant intrinsic phytase activity are used in the feed. Such microbial phytase addition allows for up to 90% of phytic acid from feed being degraded in the digestive tract of pigs and poultry [11]. Similarly, purified phytase application in a bioreactor enabled for a reduction of phytic acid content of wheat flour by up to 80% [14]. In human nutrition, the bread making recipe appears to be of particular importance. For example, rye is known to have up to fivefold higher phytase activity than common wheat [8,15], while spelt has similar phytase activity than wheat [9]. By contrast, barley, corn and rice have lower phytase activity, roughly half of that of wheat [8,9]. Additionally, prolonged dough proofing time, especially with the use of sourdough, seems to greatly reduce phytic acid content in bread between 50 and 95% compared with whole grain flour [8,16,17], which leads to significantly improved mineral absorption, at least in experiments on mice [17]. It is also well known from studies on pigs that a reduction in dietary phytic acid content leads to increased mineral uptake [11].

Nonetheless, to the best of our knowledge, no study is available in literature about mineral and phytate contents as well as phytase activities in alternative wheat species such as spelt (*Triticum aestivum* subsp. *spelta*), emmer (*Triticum turgidum* subsp. *dicoccum*) and einkorn (*Triticum monococcum*), especially when considering that studies comparing different wheat species should include a representative number of varieties per species grown under diverse environments. In particular, it is of high interest to determine differences in phytic acid content and phytase activities between varieties of a species, and to find out how large are these differences compared to the reduction possibilities using bread making recipes. Literature on the effect of bread making technologies on phytic acid content are either based on rather theoretical than applied bread making recipes or recent bread making technologies such as long yeast fermentation or popular used modifications in sourdough fermentation where not regarded. Therefore, we carried out several series of trials in which numerous varieties of the species common wheat, spelt, emmer and einkorn were grown under various environmental conditions. Whole grain flours made from harvested samples were examined to determine mineral and phytic acid content as well as phytase activity. Additionally, for some of the samples, breads were baked applying different recipes with sourdough and long yeast proofing and analyzed accordingly. We finally determined the content of FODMAPs (fermentable oligo-, di- and monosaccharides and polyols) and acrlyamide in the bread crusts, as long dough proofing times and/or the use of sourdough are reported to reduce these potential negative compounds [18,19,20].

Our objectives were to (i) determine contents of minerals, phytic acid and phytase activity within and across different wheat species, (ii) evaluate the potential of actual popular bread making recipes to enhance mineral bioavailability in comparison to the choice of flour, and (iii) determine the effects of different recipes on the amount of FODMAP and acrylamide.

## 2. Results and Discussion

The chosen very robust study design allowed for the first time in literature the elaboration of potential differences in raw material, i.e., whole grain flour, for minerals and their potential bioavailability within and between four alternative wheat species. In addition, knowledge on the variability in mineral and phytic acid contents caused by bread making recipes was extended to recent popular fermentation practices with long yeast fermentation and popular modifications in sour dough recipes.

### 2.1. The Mineral Contents of Whole Grain Flours Differ Considerably

**Figure 1:**
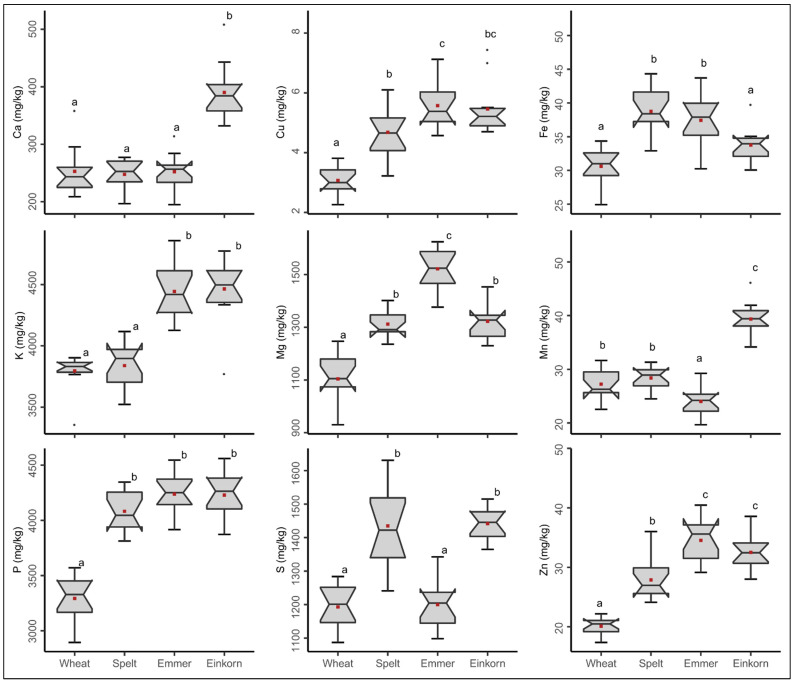
Mineral content in whole grain flours of 12 different varieties in each of the species common wheat, spelt, emmer and einkorn. Boxes with different letters indicate significant differences between the wheat species (ANOVA with Tukey’s test, *p* < 0.05). Each box summarizes the content of 36 samples (12 varieties grown at three locations in trial series 1). Mean values for each species are highlighted in red color.

We identified large differences in the content of all nine minerals measured, both within and between species (Figure 1). For instance, the amount of Fe varied across the 12 common wheat varieties in trial series 1 from 24.9 to 34.4 mg/kg with a mean of 30.6 mg/kg of whole grain wheat flour. Thus, the variety with the highest Fe content had 38% more Fe than the variety with the lowest content. When the mean values of the species were compared, common wheat had the lowest Fe content, while spelt had the highest Fe content with 38.7 mg/kg whole grain flour, a difference of 26%. Similar trends were observed for the other minerals and for simplification of reading we further focus on Fe. Considering all minerals together, spelt and especially emmer and einkorn contained significantly more minerals in whole grain flour than common wheat, which is already considered an important source of minerals in human nutrition. This large variability confirms first studies investigating mineral content in modern and old varieties from common wheat [3], and extends this knowledge to alternative wheat species spelt, emmer and einkorn tested in a robust sample setting across representative sets of (1) varieties per species and (2) field environments.

**Figure 2:**
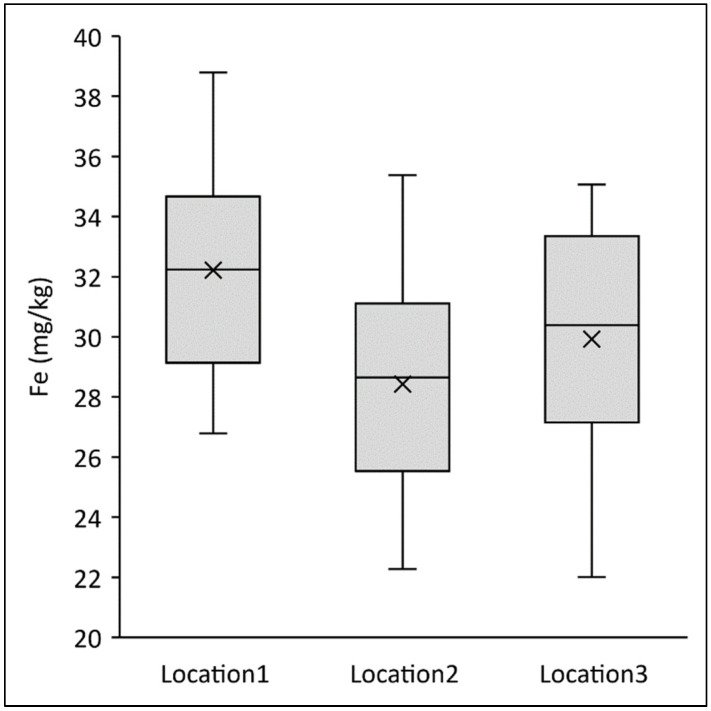
Fe content (mg/kg) in whole grain flours of 12 different varieties of common wheat grown at three different farmer locations (trial series 1).

In addition to these observed differences, wheat grain constituents always depend on the growing environment [21]. For example, the 12 common wheat varieties had an average of 32.2 mg of Fe per kg whole grain flour at the first cropping location, 28.4 mg/kg at the second and 29.9 mg/kg whole grain flour at the third growing location (Figure 2), despite the fact that cultivation practices were the same at all locations. However, soil differences and climatic conditions such as temperature and rainfall lead to very different availability of nutrients for the plants, environmental conditions that a farmer can influence only to a very limited extent [22]. Conversely, individual varieties at each location showed similar rankings for mineral content. Thus, our study underlines that by choosing a variety within a species or by choosing a different species, it is possible to increase the mineral content in the supply chain, even if the absolute values depend on the specific growing conditions at each farming location. In particular, the use of specific einkorn varieties would increase mineral content in flour sold to bakers as compared with using common wheat.

### 2.2. Phytic Acid Contents and Phytase Activity in Whole Grain Flour

The content of phytic acid can be used as a proxy to estimate the bioavailability of minerals in whole grain flours of different varieties and wheat species and the breads baked from them by different recipes. As not enough sample material was left from trial series 1 for these analyses, we performed additionally two further trial series. In trial series 2, eight varieties each of common wheat, spelt, emmer and einkorn were grown at three different locations and harvested samples were investigated on phytic acid content and phytase activity in flours and breads (only for common wheat) made thereof in three different recipes. In trial series 3, three different spelt varieties were grown at three different locations and mineral and phytic acid content were determined in flours and breads made thereof in five different recipes.

**Figure 3:**
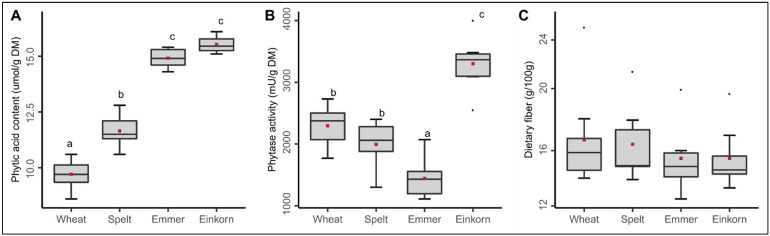
Phytic acid content (**A**), phytase activity (**B**) and total dietary fiber content (**C**). Boxes with different letters indicate significant differences between the wheat species (ANOVA with Tukey’s test, *p* < 0.05). Each box summarizes the content of 24 samples (eight varieties grown at three locations in trial series 2). Mean values for each species are highlighted in red color.

In the flours of trial series 2, we found a considerably large variance in phytic acid content within and between species (Figure 3A).

While phytic acid content varied only by about 2 μmol/g dry matter (DM) between varieties within a species, the differences across species were larger and statistically significant from each other. As with mineral content, emmer and einkorn in particular had significantly higher contents of phytic acid. For instance, common wheat had on average a phytic acid content of 9.7 μmol/g DM while emmer and einkorn an average of 14.9 and 15.5 μmol/g DM, respectively (Figure 3A). Thus, these species have more minerals, but as they are likewise bound in the phytic acid complex, they are only partially available to humans requiring strategies for degrading phytic acid in their products.

It is already known from the literature that phytic acid is partially degraded during dough proofing in the bread making process and that this depends, among other things, on the activity of the enzyme phytase [8]. Therefore, we additionally determined the phytase activity in the flours of trial series 2. Variance in phytase activity was also found between varieties within a species and across species with approximately similar magnitude (Figure 3B). For instance, phytase activity varied within the eight varieties of common wheat between 1770 and 2730 mU/g DM with an average of 2295 mU/g DM, while this variation ranged for einkorn from 2550 to 4000 mU/g DM with an average of 3300 mU/g DM. Dinkel and emmer had slightly lower phytase activity than common wheat on average. However, the extent to which the elevated phytase activity of einkorn can compensate for the higher phytic acid content in einkorn flour must be shown by further studies. The values for common wheat and spelt are comparable to those reported in literature [9]. However, all wheat species tested in our study had a three to fourfold lower phytase activity than reported for rye [8,9], which could be experimentally proven in this study at least for the commercial whole grain rye flour (7800 mU/g DM) used for baking in trial series 2.

### 2.3. Phytic Acid Contents in Breads Prepared with Different Recipes

**Figure 4:**
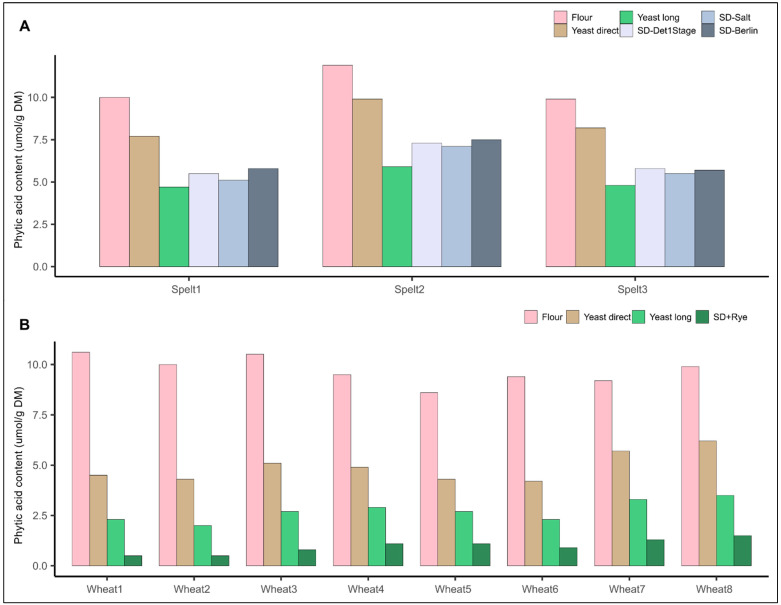
Phytic acid content in whole grain breads from three spelt varieties baked with five different recipes ((**A**), trial series 3) and eight common wheat varieties baked with three different recipes ((**B**), trial series 2). SD-Det1Stage = Detmolder 1 stage sourdough, SD-Salt = Monheim salty sourdough, SD-Berlin = Berlin short sourdough, SD+rye = Detmolder 1 stage sourdough with standard rye flour.

To compare the influence of varietal choice within a species on phytic acid content with the influence of different recent bread making recipes, we undertook two series of bread baking experiments. First, we baked three different spelt varieties with five different recipes (trial series 3): two yeast–based recipes popular among German bakers and three different and popular sourdough recipes. All bread making recipes considerably reduced the phytic acid content of the breads (Figure 4A, Table 1). The most significant reduction of phytic acid content was observed using the yeast long proofing recipe in all three spelt varieties with a reduction of phytic acid content by >50% compared to the initial whole grain flour, followed closely by the three sourdough recipes. The difference in phytic acid content when comparing the flours or breads of the three different spelt varieties was considerably smaller than the difference in phytic acid content when comparing the flour with the baked breads.

As only three spelt varieties were used, which might not fully represent the variability within a species, we additionally compared phytic acid content in flours and breads made from eight varieties of common wheat in a further trial series, but only with three bread making recipes. We used the two yeast-based recipes from trial series 3, but only one sourdough recipe (modified Detmolder 1 Stage sourdough). Similar to the above-mentioned trial with spelt, bread making lowered the final phytic acid content in breads significantly and this reduction was more important than the choice of a common wheat variety with possibly lower phytic acid content or higher phytase activity (Figure 4B). In particular, compared to the initial whole grain flour, phytic acid content was reduced by 50 and almost 75% via short and long yeast fermentation, respectively. This confirms several studies, which have shown the potential to reduce phytic acid content in breads via different bread making recipes [16,17,23]. Furthermore, the long yeast proofing recipe was shown to be able to largely degrade phytic acid in two different trial series, which has not been described yet in literature.

In a final step, we designed an optimized sourdough baking protocol. The Detmolder 1 stage sourdough method was used as basis, but instead of 15% of the flour, 25% of the flour was acidified and this 25% of flour was not the variety-specific common wheat flour, but a standard whole grain rye flour. The idea behind this modification was that rye, due to its significantly higher phytase activity compared to common wheat, contributes to faster or higher phytic acid degradation. Indeed, in all eight whole grain wheat breads, phytic acid was mostly degraded using this optimized sourdough protocol with phytic acid contents below 10% compared to whole grain wheat flour (Figure 4B). This finding supports the notion that recipes and the dough management by the bakers are of pivotal importance to increase bioavailability of minerals such as Fe and Zn in whole grains for the consumers. Moreover, the degradation of phytic acid seems to not necessarily require specific baking technologies, which might be difficult to implement in practice. It can rely on the smart combination of raw material, e.g., rye as sourdough starter, and popular fermentation processes, e.g., overnight yeast fermentation or Detmolder 1 stage sour dough.

### 2.4. Mineral Contents in Bread Compared to the Flour Used in the Recipe

We compared mineral contents in whole grain flour with those in the bread made from these flours (Table 1). The breads contained at least the same amount of minerals as the flours. We also found no significant difference in the mineral content between the two yeast and three sourdough recipes. By contrast, differences were detectable between the whole grain flours (three different spelt varieties) and the breads baked from them. For instance, Fe content in whole grain flours increased in the order spelt variety 3-spelt variety 1-spelt variety 2. Taking average values of all breads baked from an individual variety, showed the same order of magnitude in Fe content of the different spelt varieties. This underlines that the extraction protocol and chemical analyses worked well in the different matrices flour and bread and shows that the selection of varieties with higher mineral content led to bread with higher mineral content, which are then also available to humans using appropriate bread making recipes.

### 2.5. The Potential of Bread Making Recipes to Maximize Bread Quality

In addition to the discussed impact on phytic acid content, previous studies have additionally shown that bread making recipes can positively alter bread properties or ingredients discussed to impact human health, e.g., FODMAPs (fermentable oligo-, di- and monosaccharides and polyols), acrylamide or allergenic proteins. For instance, compared to direct yeast proofing, long yeast proofing results in higher freshness and better flavor in the bread while baking volume is slightly reduced in some cases [24,25], which is also reported from bakers’ practical experience. In addition, the abundance of at least 20% of all proteins measurable in bread today are influenced by bread making recipes [26]. However, the abundance of potential allergenic proteins depended more on the choice of the cereal species than the recipe applied for making breads in that study.

By contrast, the content of FODMAPs in breads are strongly influenced by dough management [18,25], which could be confirmed in our study (Table 1). Interestingly, sourdough recipes led to significant lower FODMAP contents of the breads compared to yeast fermentation, which also confirms previous studies [27]. FODMAPs are discussed to have a negative impact on patients with irritable bowel syndrome and/or non-gluten wheat sensitivity [18,20,25,27]. However, a recent human study with patients related to these diseases showed lowest reactions in an irritable bowel disease score when eating gluten-free breads with a high amount of added FODMAPs as compared to a gluten-free diet or diets with breads made from spelt and wheat with low FODMAP content due to long dough proofing times [28]. Thus, more research is warranted to elaborate the impact of FODMAP content on human health.

Acrylamide is assumed to be carcinogen and can occur when starch containing food is highly heated, e.g., potato crisps, potato fries, coffee but also gingerbread, crispbread or crusts of dark baked breads [19,29]. The amount of acrylamide in bread crusts was shown to be reduced with prolonged yeast proofing time [19], which could be confirmed with our study. In particular, long yeast proving led by far to lowest acrylamide concentrations in bread crusts, while sourdough recipes resulted in higher acrylamide contents in bread crusts (Table 1), which is to the best of our knowledge not yet reported in literature. As the bread crust makes only a small percentage of a normal bread, acrylamide content of whole breads is mostly considerable below under EU thresholds, but the possibility to further reduce acrylamide in bread via dough proofing time should be kept in mind.

Summarizing, we could extent the knowledge of the large potential of different bread making recipes on the reduction of potential negative ingredients in bread, e.g., phytic acid, FODMAPs and acrylamide. It would be desirable that these possibilities are further investigated. In particular, more human studies are urgently required to elaborate the impact of all these possibilities based on well-defined study material on healthy humans but also humans suffering hidden hunger or other diseases [10].

### 2.6. Outlook: Collaboration along the Supply Chain Is Mandatory for Improving Future Nutrition

We elaborated in an extensive and robust sample setting that mineral content varies greatly depending on the type of the chosen cereal and even within a given cereal on the chosen variety. Furthermore, phytic acid degradation indicated that the availability of minerals is influenced in particular by the baker’s recipe and dough management and that a smart combination of raw material with recently popular fermentation techniques appears as promising strategy. This exemplary research along the supply chain has shown that for healthier nutrition of the world’s population, for example to reduce hidden hunger or to establish the personalized nutrition, it is necessary to think and act in a more holistic way. A simplified supply chain for wheat bread starts with the breeding of a new wheat variety that a farmer grows, the miller processes, and the baker transforms into the final product that humans then consume. All stakeholders in that supply chain have an impact on the amount of minerals such as Fe that would ultimately be available to humans in bread. First of all, the raw material, e.g., a batch of wheat grains, must contain as much Fe as possible, which can be influenced by the choice of variety. At the miller, despite the choice of variety, cereal grains with different mineral contents arrive depending on where they are grown. This can hardly be influenced, but the mineral content should be measured with the help of rapid tests yet to be established. In addition, the minerals should not be “milled out”. Moreover, mineral content is lower in the refined flour as compared to whole grain flour. Finally, the baker must also choose suitable recipes, such as a long yeast proofing or a sourdough starter with whole grain rye flour, so that minerals such as Fe and Zn from whole grains are available to humans in the bread. To our knowledge, little of all these recommendations are considered in the supply chains so far. For example, in wheat, quality enhancement is thought to be achieved only by maximizing the bread loaf volume using refined flours.

## 3. Materials and Methods

Field trials were performed for all trial series at following locations: Stuttgart-Hohenheim (48°42′50″ N, 9°12′58″ E, 400 m above sea level, 8.5 °C mean average temperature, 685 mL mean annual precipitation, soil type: silty clay), Oberer Lindenhof (48°28′26″ N, 9°18′12″ E, 700 m above sea level, 6.8 °C mean average temperature, 924 mL mean annual precipitation, soil type: silty clay), and Eckartsweier (48°31′45″ N, 7°51′18″ E, 141 m above sea level, 9.9 °C mean average temperature, 726 mL mean annual precipitation, soil type: loamy sand). The examined samples were taken from breeding trials, which consisted for each species of few important varieties of the market and dozens of new breeding lines from the breeding program of the State Plant Breeding Institute of the University of Hohenheim. The wheat species were investigated in separate but adjacent trials at individual locations using an un-replicated field design randomized separately for each species across test locations. All trials received the same field treatments of intensive conventional farming practices except for nitrogen (N) fertilization, where common wheat, spelt, emmer and einkorn were fertilized to reach the following level of N including N measured in the soil (“Nmin”): 180, 160, 60 and 60 kg N/ha, respectively. This individual adjustment was performed to reflect recent agricultural practice in conventional production. Furthermore, owing to its field resistance to fungal diseases einkorn did not receive any fungicide treatment in contrast to all other species. Furthermore, growth regulators and herbicides were applied twice but no irrigation was performed.

In trial series 1, 12 varieties of each of the species namely common wheat, spelt, emmer and einkorn were grown at three different locations in unreplicated trials and the mineral content of their whole grain flours was determined in mg/kg by ICP-OES after microwave-assisted digestion without technical replication in the lab [30]. The most important varieties in the German market were taken and completed to sum up to 12 in the case of spelt, emmer and einkorn with few late breeding lines from the breeding program of the State Plant Breeding Institute of the University of Hohenheim representing recent variability in these species in the German flour market. As not enough sample material was left from trial series 1 for analyzing phytic acid content and phytase activity in flours and breads made thereof, we performed additionally two further trial series.

In trial series 2, eight varieties each of common wheat, spelt, emmer and einkorn were grown at three different locations in unreplicated trials. A selection of varieties were taken as in trial series 1. Representative composite samples of each variety were then prepared from the harvest samples of the three locations, i.e., taking the same amount of seeds per harvest location and mix them intensively before further quality analyses. The following traits were recorded on the whole grain flour of these mixed samples: content of total dietary fiber in g/100 g by AOAC 991.43, which determines cellulose, beta-glucan, galactomannan, pectin, arabinoxylan, arabinogalactan and partly fructo-oligosaccharides/inulin, resistant starch and oligosaccharides (without technical replication in the lab), content of phytic acid (InsP6) in µmol/g DM by chromatography according to Zeller et al. [31], and phytase activity in mU/g DM by direct incubation according to Greiner and Egli [23], the latter two both with two technical replicates in the lab. As the mean and range of dietary fiber was similar across the four wheat species (Figure 3C), we mainly focused on minerals and their bioavailability.

From the eight common wheat variety samples of the trial series 2, bread was baked by an experienced professional baker and baking trainer using three different recipes and the phytic acid content was determined in the breads. The following recipes were used: 1. direct yeast proofing with 500 g whole grain flour, 450 g water, 10 g salt and 15 g yeast. The doughs were kept at room temperature for three hours from the starting time to the time when the finished bread was put in the oven; 2. long yeast proofing with 500 g whole grain flour, 450 g water, 10 g salt and 5 g yeast. In this case, the doughs were prepared the day before, put into the refrigerator at 4 °C for 18 h, and then left at room temperature for another three hours from the time of removal from refrigeration until the finished shaped bread was put in the oven; 3. Sourdough (SD) with rye according to the Detmolder 1-stage sourdough method with acidification of 25% of the flour quantity, where this 25% was a standard whole grain rye flour with a determined phytase activity of 7800 mU/g DM and phytic acid content of 9.5 µmol/g DM. Specifically, 125 g of whole grain rye flour, 100 g of water, 7 g of rye sourdough starter were mixed and allowed to rise at room temperature for 18 h. On top of this, 225 g of sourdough, 375 g of whole grain wheat flour, 350 g of water, 10 g of salt and 10 g of yeast were kneaded and left for three hours at room temperature until the shaped breads were put into the oven.

In trial series 3, three very popular spelt varieties were grown at three locations in unreplicated trials. Representative composite samples of each variety were then prepared from the harvested samples of the three locations as described for trial series 2 and whole grain flour was processed for analyzing contents of minerals and phytic acid without technical replication in the lab. Sugar alcohols, mono-, di- and oligosaccharides were determined in mg/100 g by anion exchange chromatography with pulsed amperometric detection (sugar alcohols, mono- and disaccharides adapted from Thermo Fisher Scientific Technical Note 72225; oligosaccharides according to Thermo Fisher Scientific Application Note 1149) after aqueous extraction without technical replication in the lab [21]. The sum of all measured oligosaccharides, sugar alcohols and excess fructose was taken as FODMAP content. In addition, the amount of acrylamide in µg/kg in the crust of the test breads was analyzed according to Rosen and Hellenäs without technical replication in the lab [32].

Finally, bread was baked from the whole-grain flours by an experienced professional baker and baking trainer using five different recipes and the contents of minerals and phytic acid were measured. The following recipes were used: 1. direct yeast proofing with 500 g whole grain flour, 350 g water, 10 g salt and 15 g yeast. In this case, the doughs were kept at room temperature for three hours from the starting time until the time when finished shaped bread was put into oven; 2. long yeast proofing with 500 g whole grain flour, 350 g water, 10 g salt as well as 5 g yeast. In this case, the doughs were prepared the day before, put into the refrigerator at 8 °C for 18 h and then left at room temperature for another three hours from the time they were taken out of refrigerator until the finished shaped bread was put into oven; 3. Detmolder 1 stage sourdough with acidification of 15% of the flour quantity. For this, 75 g of whole grain spelt flour, 75 g of water, 7.5 g of spelt sourdough starter were mixed and left to rise at room temperature for 18 h. Then, 150 g of sourdough, 425 g of whole grain spelt flour, 275 g of water, 10 g of salt and 10 g of yeast were kneaded and left for three hours at room temperature until the formed bread was put into oven; 4. Monheim salty sourdough with acidification of 15% of the flour quantity. For this purpose, 75 g of whole grain spelt flour, 75 g of water, 15 g of spelt sourdough starter, 1.5 g of salt were mixed and left to rise for 18 h at a temperature that dropped from 35 °C to 25 °C. The sourdough was then leavened for three hours at room temperature. Then 150 g of sourdough, 425 g of whole grain spelt flour, 275 g of water, 8.5 g of salt and 10 g of yeast were kneaded and left for three hours at room temperature until the shaped breads were put into oven; 5. Berlin short sourdough with acidification of 15% of the amount of flour. For this purpose, 75 g of whole spelt flour, 75 g of water, 15 g of spelt sourdough starter were mixed and allowed to rise for four hours at 35 °C. Then 150 g of sourdough, 425 g of whole grain spelt flour, 275 g of water, 10 g of salt and 10 g of yeast were kneaded and kept for three hours at room temperature until the shaped breads were put into oven.

Trial series 1 was analyzed according to mixed linear models in a one-way ANOVA. For this purpose, the statistical program R [33] was used with the aid of the package ASREML [34] to calculate varietal mean values as best linear unbiased estimates (BLUEs) assuming genotype as the fixed effect in the statistical model. For trial series 2 and 3 composite samples from the individual varieties were used and, thus, only direct comparisons or grouped comparisons via *t*-test were performed.

## Figures and Tables

**Table 1:** Contents of phytic acid (μmol/g), minerals (mg/kg) and FODMAPs (mg/100 g) in whole grain flours and breads baked from flours of three spelt varieties (trial series 3) as well as acrylamide in bread crusts (μg/kg; trial series 3; SD-Det1Stage = Detmolder 1 stage sourdough, SD-Salt = Monheim salty sourdough, SD-Berlin = Berlin short sourdough).

Material	Variety	Phytic Acid	Ca	Cu	Fe	K	Mg	Mn	P	S	Zn	FODMAPs	Acrylamid
Flour	Spelt1	10.0	223.8	4.0	30.0	4262.9	1007.3	24.3	3590.7	1079.9	17.1	4210.3	NA
Flour	Spelt2	11.9	218.2	5.0	39.8	4316.7	1165.5	27.9	3939.7	1249.1	20.3	3360.3	NA
Flour	Spelt3	9.9	254.8	4.1	27.7	4241.1	940.8	20.4	3451.3	1078.4	18.4	3692.3	NA
Direct yeast	Spelt1	7.7	331.9	4.1	39.5	4571.3	1082.8	25.1	3931.0	1226.5	21.0	3252.7	346
Long yeast	Spelt1	4.7	316.5	3.9	36.6	4383.5	1047.2	24.6	3797.4	1258.9	18.5	3462.2	89
SD-Det 1Stufe	Spelt1	5.5	319.7	4.1	32.9	4511.0	1068.6	25.1	3867.2	1226.1	20.5	2443.9	442
SD-Salt	Spelt1	5.1	318.0	4.1	32.3	4557.6	1058.1	24.7	3869.9	1232.2	20.4	2551.7	440
SD-Berlin	Spelt1	5.8	323.6	3.9	33.6	4540.1	1063.6	25.0	3875.7	1205.7	20.2	2646.6	336
Direct yeast	Spelt2	9.9	319.9	5.3	41.9	4579.6	1212.1	28.2	4232.8	1429.2	24.3	2598.2	290
Long yeast	Spelt2	5.9	321.6	5.6	42.0	4462.6	1208.6	28.4	4171.9	1471.4	22.5	3103.4	39
SD-Det 1Stufe	Spelt2	7.3	315.7	5.3	41.5	4589.3	1221.6	28.5	4253.4	1432.2	23.6	2196.6	344
SD-Salt	Spelt2	7.1	319.4	5.3	41.3	4586.2	1217.8	28.2	4248.7	1469.4	23.9	2161.3	224
SD -Berlin	Spelt2	7.5	314.4	5.3	40.2	4498.1	1174.0	27.6	4127.3	1440.3	23.7	2214.1	274
Direct yeast	Spelt3	8.2	360.2	4.7	31.1	4700.8	1035.7	21.8	3848.7	1243.7	23.1	2987.7	440
Long yeast	Spelt3	4.8	357.8	4.6	32.0	4551.1	1011.8	21.7	3750.5	1304.6	20.4	3098.7	97
SD-Det 1Stufe	Spelt3	5.8	360.3	4.8	31.9	4654.6	1047.2	22.3	3852.5	1269.0	23.5	2239.2	524
SD-Salt	Spelt3	5.5	348.6	4.4	29.6	4557.3	996.6	21.0	3715.6	1230.5	21.6	2318.2	594
SD-Berlin	Spelt3	5.7	359.6	4.6	31.5	4786.2	1061.5	22.5	3933.5	1245.2	22.5	2419.4	752
Mean flour		10.6	232.3	4.4	32.5	4273.5	1037.8	24.2	3660.6	1135.8	18.6	3754.3	NA
Mean bread		6.4	332.5	4.7	35.9	4568.6	1100.5	25.0	3965.1	1312.3	22.0	2646.3	NA
*t* test		0.01	0.01	0.48	0.47	0.00	0.45	0.76	0.16	0.07	0.05	0.03	NA
Mean yeast bread		6.3	334.7	4.7	37.2	4541.5	1099.7	25.0	3955.4	1322.4	21.6	3083.8	216.7
Mean SD bread		6.9	331.0	4.7	35.0	4586.7	1101.0	25.0	3971.5	1305.6	22.2	2354.5	436.7
*t* test		0.45	0.73	0.88	0.40	0.42	0.98	0.99	0.88	0.77	0.57	0.00	0.03

## Data Availability

Data is contained within the article.
